# Purification of Messenger Ribonucleoprotein Particles via a Tagged Nascent Polypeptide

**DOI:** 10.1371/journal.pone.0148131

**Published:** 2016-01-25

**Authors:** Diana P. Inchaustegui Gil, Christine Clayton

**Affiliations:** Zentrum für Molekulare Biologie der Universität Heidelberg (ZMBH), DKFZ-ZMBH Alliance, Heidelberg, Germany; University of Toronto, CANADA

## Abstract

The cytoplasmic fates of mRNAs are influenced by interactions between RNA-binding proteins and *cis* regulatory motifs. In the cytoplasm, mRNAs are present as messenger ribonucleoprotein particles, which include not only proteins that bind directly to the mRNA, but also additional proteins that are recruited via protein-protein interactions. Many labs have sought to purify such particles from cells, with limited success. We here describe a simple two-step procedure to purify actively translated mRNAs, with their associated proteins, from polysomes. We use a reporter mRNA that encodes a protein with three streptavidin binding peptides at the N-terminus. The polysomal reporter mRNA, with associated proteins, is purified via binding to a streptavidin matrix. The method takes four days, and can be applied in any cell that can be genetically manipulated. Using *Trypanosoma brucei* as a model system, we routinely purified 8% of the input reporter mRNA, with roughly 22-fold enrichment relative to un-tagged mRNAs, a final reporter-mRNA:total-mRNA ratio of about 1:10, and a protein purification factor of slightly over 1000-fold. Although the overall reporter mRNP composition is masked by the presence of proteins that are associated with many polysomal mRNAs, our method can be used to detect association of an RNA-binding protein that binds to specifically to a reporter mRNA.

## Introduction

The first attempts to purify specific polysomal mRNAs were made forty years ago. Antibodies to a protein of interest were used to immunoprecipitate the nascent polypeptide, and the associated mRNAs were purified for use in *in vitro* translation, as probes for gene or cDNA libraries, or for direct cDNA cloning [1–6]. These initial attempts focussed on cells that were highly specialised for production of a few products, which greatly facilitated purification. The method rapidly fell out of use in favour of more convenient and versatile methods such as differential hybridisation and expression cloning [7].

More recently, interest in purification of specific RNAs has revived, but this time with the purpose of identification of bound proteins from native ribonucleoprotein complexes (RNPs). So far, all reported methods have used the RNA as the target for purification [8]. Hybridisation to complementary oligonucleotides can be used to isolate abundant, stable ribonucleoprotein complexes (RNPs) [9]. For example, hybridisation with large sets of biotinylated oligonucleotides, followed by SILAC quantitative mass spectrometry, was also recently used to identify proteins that cross-linked to the Xist RNA [10,11]. Expression of transgenic RNAs bearing specific affinity tags is an alternative to hybridisation. Two types of aptamers are in common use: those that bind to small molecules, and those that are bound in a highly specific fashion by proteins such as MS2 coat protein, the U1A protein and the lambdaN peptide [12]. Several labs have been able to demonstrate specific purification of RNAs, and also, by Western blotting, the co-purification of proteins that were already known to bind to those mRNAs. For example, a tobramycin-binding aptamer was used to isolate the U2 snRNP [13]. Slobodin and Gerst [14] purified yeast and mammalian mRNAs bearing the MS2 aptamer, with a co-expressed fusion protein consisting of the MS2 coat protein (to bind the mRNA), GFP (for visualization) and streptavidin binding peptide (for purification). They showed clear specific purification of several tagged mRNAs, expressed at endogenous levels, by real-time RT-PCR, and could also demonstrate sequence-specific co-purification of known mRNP proteins by Western blotting. The highest yield reported so far—of 4.5%—involved mRNPs assembled on a reporter mRNA bearing streptavidin-binding aptamer [8].

None of the methods described so far has been shown to be suitable for characterisation of native mRNP proteomes by mass spectrometry, because the purification was insufficient to enable detection of specifically bound proteins above the background contamination. In this paper, we describe a two-step procedure. We first purify polysomes, then enrich the mRNA of interest via streptavidin-binding tags on the nascent polypeptide. We achieved better purification that the previously-described methods for mRNA, and could show specific protein association, but the purity and yield were once again insufficient to enable characterisation of an individual mRNP by mass spectrometry.

## Results

### Development of the strategy

**Fig 1:**
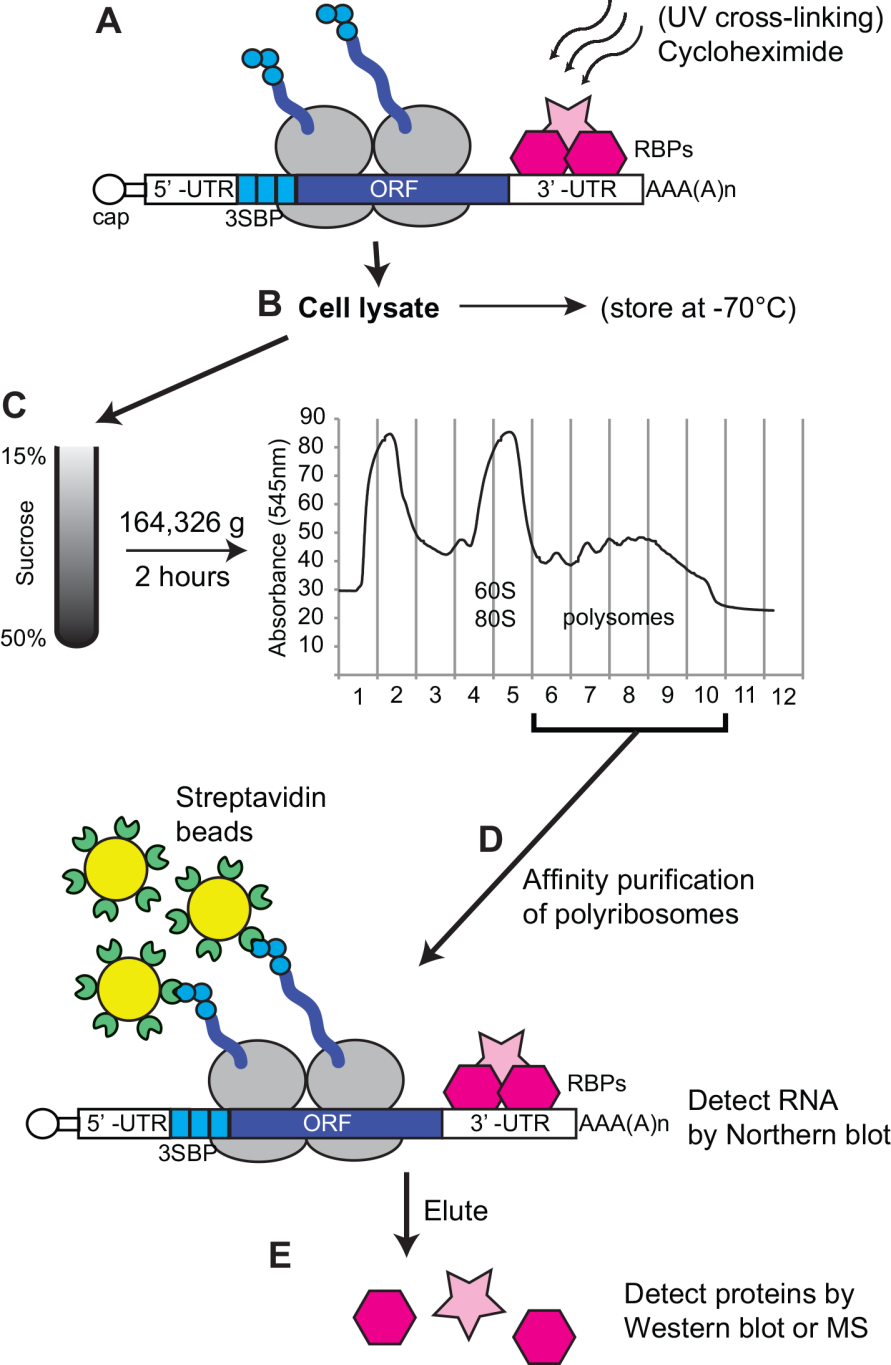
Work-flow for the purification. RNA and proteins were UV-cross linked at 254nm followed by 5 min of incubation with cycloheximide (CHX). Cells were lysed with detergent and the cleared lysate loaded onto a sucrose gradient (15–50% sucrose). The fractions containing actively translating polyribosomes were pooled and incubated with streptavidin sepharose beads for 1 h. The success of the purification was assessed by Northern blot. Proteins present in the purified mRNPs were detected by mass spectrometry (MS) or Western blot. SBP: streptavidin binding protein; RBPs: RNA binding proteins; UTR: untranslated region; ORF, open reading frame; CHX, cycloheximide; MS, mass spectrometry.

To develop the strategy, we designed a reporter mRNA. It encodes a chloramphenicol acetyltransferase (CAT) protein that has 3 streptavidin-binding peptides (SBPs) at the N-terminus [15] and -SKL at the C-terminus (Fig 1A). The C-terminal -SKL tripeptide should target a protein to microbodies, such as peroxisomes in mammalian cells or glycosomes in trypanosomes. An mRNA that is identical, apart from the absence of SBPs from the encoded CAT protein, served as a negative control. For use in trypanosomes, the plasmid was integrated into the genome and the mRNA was expressed from a tetracycline-inducible promoter. For use in other systems, the open reading frame could be excised from the plasmid using unique restriction sites. Our procedure involves cell lysis (step B), purification of polysomes on sucrose gradients (step C), affinity purification on streptavidin beads with washing (step D), and elution of the proteins from the beads (step E). We tested numerous variations of this procedure. A very detailed final protocol is included as S1 Text and S1 Fig and a discussion of the variations is in S2 Text.

**Fig 2:**
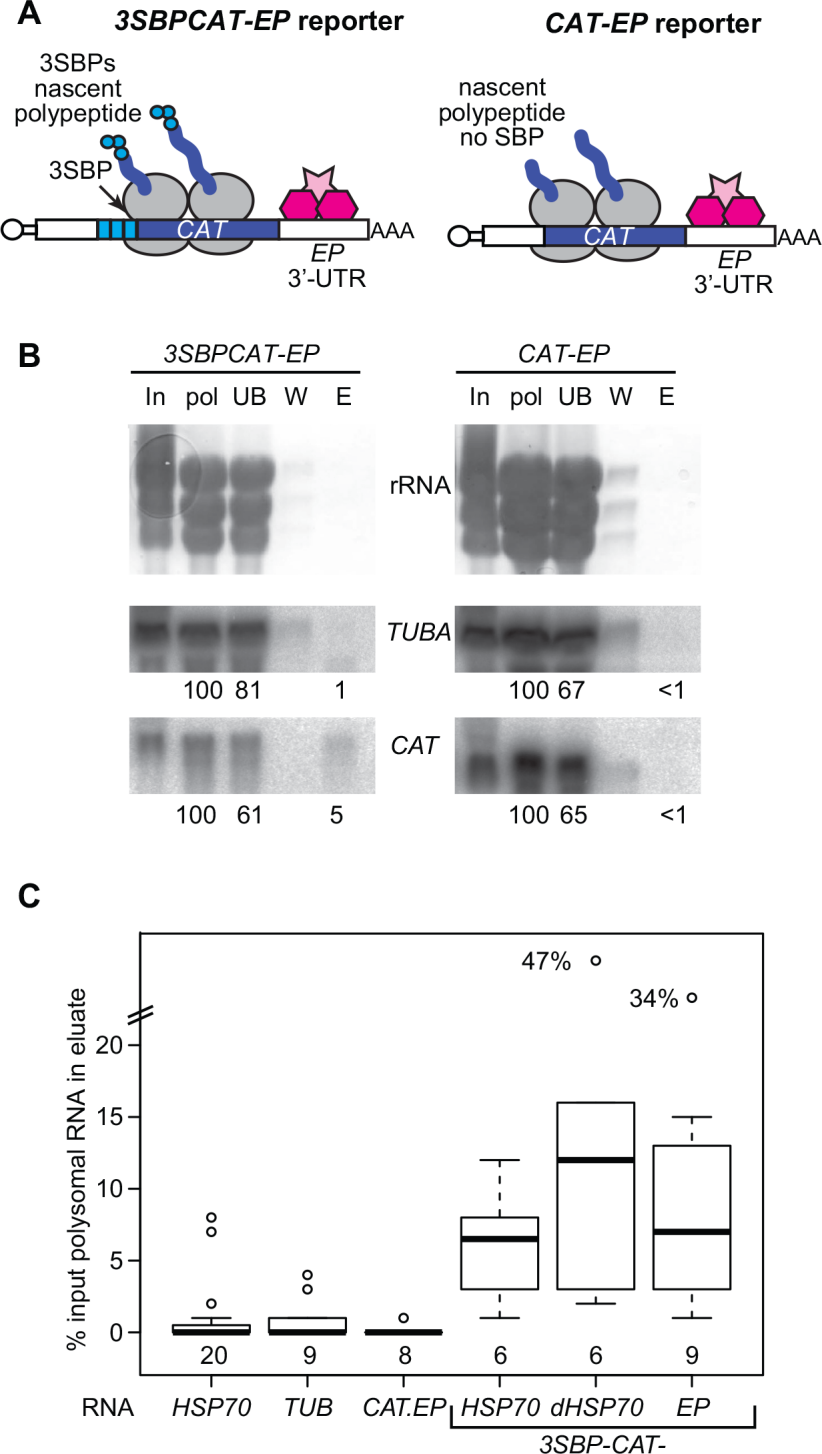
Purification of the *3SBP-CAT-SKL-EP* mRNP. (A) The mRNAs. Multi-tag reporter (upper left) composed of 3SBPs at the N-terminus of the ORF and the control reporter (upper right), without the 3SBPs. SBP: streptavidin binding peptide; UTR: untranslated region; CAT: chloramphenicol acetyltransferase; *SL*: spliced leader; RBP: RNA-binding protein. The black portion is the *CAT* gene. (B) Northern blot showing purification of the *3SBP-CAT-SKL-EP* mRNA and failure to purify *CAT-SKL-EP* mRNA. *TUBA*: alpha tubulin. The numbers below the blots are the relative amounts of the mRNA measured, relative to the polysomal RNA input. These numbers are already correcting for loading. In: input cells (6x10^7^ cell-equivalents): pol: polysomes (6x10^7^ cell-equivalents); UB: unbound (6x10^7^ cell-equivalents); W: wash (6x10^7^ cell-equivalents); E: eluate (8x10^7^ cell-equivalents). Each probe detects a single band. (C) Box plot for all purifications similar to those in this Figure and Fig 3. The centre line is the median, the boxes extend over the 25th to 75th percentiles, and the whiskers show the 95% confidence limits. The number of independent experiments for each construct is shown beneath the boxes.

We tested numerous variations of this procedure, using the reporters shown in Fig 2A. These reporters differ only in the presence or absence of the 3SBP tag. Both have the 3'-UTR of the *EP* mRNA, which is sufficient to ensure that the mRNA is stable in procyclic forms [16,17]. The mRNAs were produced from an inducible RNA polymerase I promoter, and titration against a standard showed that there were approximately 400 copies of the mRNA per cell (not shown). It therefore constituted approximately 1% of the total mRNA. Since *EP* procyclin mRNA constitutes about 3% of the mRNA, the reporter expression level was in the physiological range.

To monitor the purifications, we protease-treated the samples and measured the yields of *CAT* mRNA, comparing the results using the reporter with and without the streptavidin binding peptides. As an internal control, we measured the amount of *TUB* (tubulin) mRNA, which is one of the most abundant mRNAs at about 1000 molecules/cell [18,19]. Since it is centrally important that the mRNA remains intact during the procedure in order to conserve mRNP integrity, and the buffers contain heparin, which inhibits reverse transcriptase, we used Northern blotting to assess purification of full-length mRNA. Results for these numerous tests are not shown but can be obtained from the authors on request.

We first tested a variety of cell lysis methods (step B). We were worried that the presence of mature 3SBP-CAT in our lysates might compromise the efficiency of polysome purification, by saturating the streptavidin matrix and thus competing with the nascent polypeptides. For this reason, the expressed reporter protein has a C-terminal signal, -SKL, which targets it to microbodies (glycosomes in trypanosomes). Indeed, isotonic breakage with silicon carbide or glass beads, followed by centrifugation at 33,000g, removed 60–80% of the CAT activity from the lysate (not shown). The use of this procedure would have the additional benefit of improving the purification by removing most glycosomal proteins. The effect on contamination with mitochondrial proteins would be more limited, since trypanosomes have a single branched mitochondrion, which runs the length of the cell and is easily broken. Unfortunately, however, only about 10% the *CAT* mRNA remained in the supernatant after the 33000g centrifugation, and large polysomes (5 ribosomes and more) were selectively lost. It is possible that polysomes are trapped in the trypanosome microtubule cytoskeleton: if so, this procedure might work better in other cell types. We therefore decided that in order to obtain polysome yields that sufficed for affinity purification and subsequent mass spectrometry, we would have to lyse the cells with mild detergent. The resulting cleared lysates were loaded onto 15–50% sucrose gradients and polysomes purified using a standard protocol (C) [20]. The polysomal fractions were then pooled and immediately subjected to affinity purification.

A large number of variations in the protocol was tried for the affinity purification step. We used a reporter bearing three N-terminal SBPs because a reporter bearing only a single SBP on the nascent polypeptide did not bind to streptavidin-coated magnetic beads. When we compared magnetic beads and sepharose affinity matrices, we found that the yield of *CAT* mRNA, relative to *TUB* mRNA, was usually 2-3-fold higher using sepharose. Using both magnetic beads and sepharose, we attempted to elute the SBP-bound polysomes using 10 mM biotin, as recommended by the matrix manufacturers, but no detectable RNA or protein was obtained. This could be because each polysome is bound to the matrix by several different nascent polypeptides, as well as by three SBPs per peptide. We also tried to elute using RNase; this test (which was monitored by Western blot) is described later in the paper. Ultimately, we settled on the use of the sepharose affinity matrix, with elution of the bound polysomal proteins by boiling in standard SDS-polyacrylamide gel sample buffer.

In Fig 1 we also show some optional steps. Before lysis, the cells can be subjected to UV cross-linking. This should achieve partial covalent linkage of proteins to mRNAs and will therefore improve co-purification of proteins bound with low affinity; it will not, however, prevent association of proteins with the mRNAs after cell lysis. We added cycloheximide to inhibit polypeptide chain elongation, since this increases the polysome yield. Prior to polysome gradient analysis, trypanosomes can be stored as pellets at -70°C and used the next day; they can also be stored in lysis buffer with 10% glycerol. We have, however, not attempted affinity purification starting with frozen material.

### Purification of the *3SBP-CATSKL-EP* mRNP

**Fig 3:**
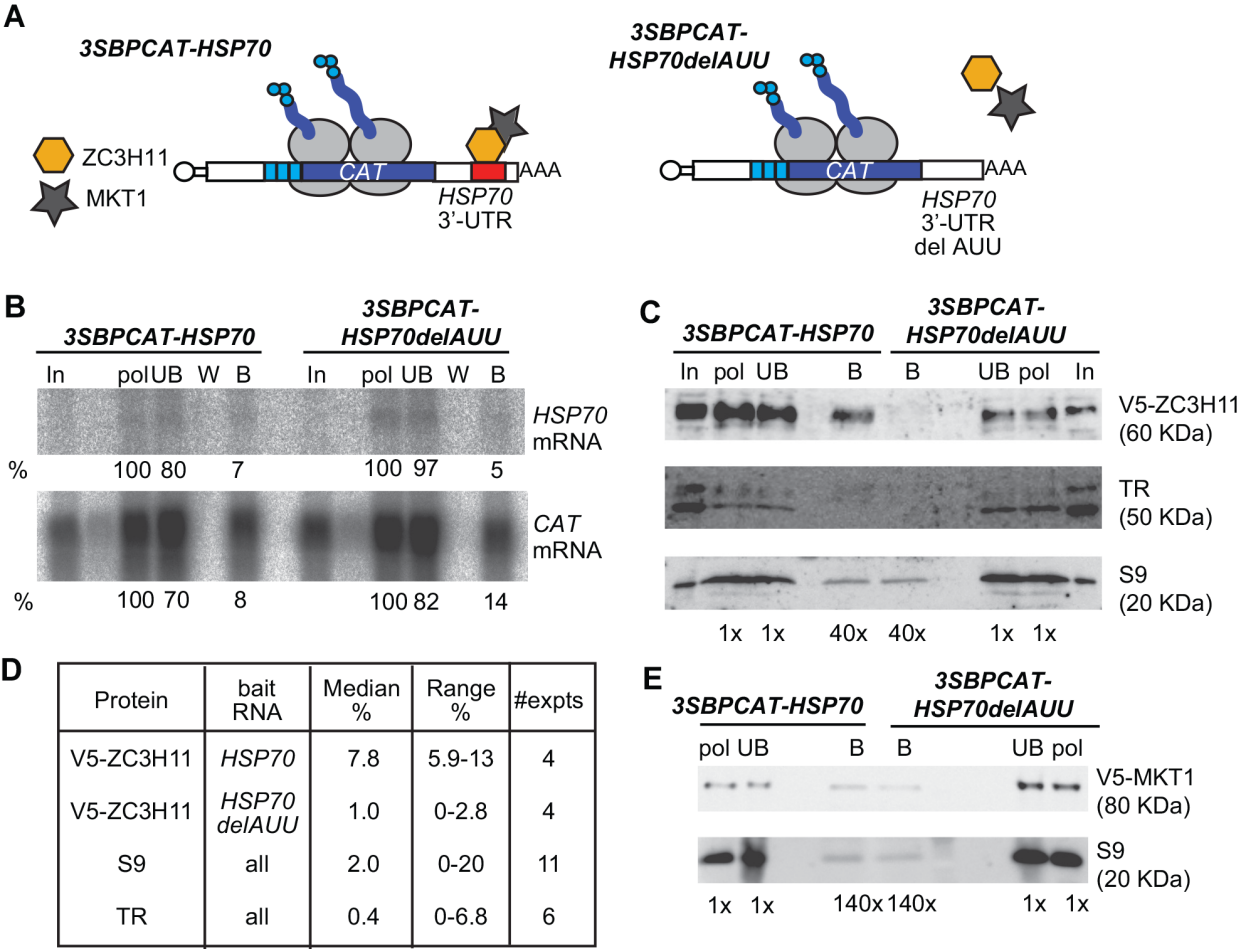
ZC3H11 co-purifies with the *HSP70* 3’ -UTR. (A) Schematic representation of the reporters used for the HSP70 mRNP purification. The control reporter (right) lacks the AUU repeat that is bound by ZC3H11. (B) Northern blot showing the purification of both *3SBP*-*CAT* mRNAs; details as in Fig 2. (C) Western blot showing specific co-purification of V5-ZC3H11 with *3SBP*-*CAT*-HSP70 mRNA. The figures below the lanes show the relative loading in cell-equivalents. For the beads, about 10^9^ cell-equivalents were loaded. TR: trypanothione reductase (with an additional cross-reacting band); S9: ribosomal protein S9 (which detects two bands). A single V5-tagged band was observed. (D) Summary for four independent purifications. Results for the *CAT* baits are for four experiments each; those for TR and S9 are for all eight experiments. (E) The method cannot be used to identify proteins that bind to many other mRNAs in addition to the target mRNA. The same experimental set-up as in (B), but in cells expressing V5-MKT1. For the beads, about 10^9^ cell-equivalents were loaded.

Results of a typical purification using our optimised protocol, with cells expressing either *3SBP-CAT-EP* or *CAT-EP* reporters (Fig 2A), and without UV-cross-linking, are shown in Fig 2B. In this experiment, approximately 5% of the input *3SBP-CAT-EP* mRNA, but no detectable *CAT-EP* mRNA, were purified. The *TUB* mRNA signal was generally much lower and sometimes undetectable in the eluates; in the instance shown it was roughly 1% of input for the *3SBP-CAT-EP* purification and undetectable for *CAT-EP*. Fig 2C summarises the results of many more purifications, using these and the reporters shown in Fig 3A. Ignoring the outliers, the median proportion of *3SBP-CAT-SKL* mRNA purified was 6.3% (range 1–16%). For *TUB* mRNA, the corresponding numbers (from the same purifications) were median 0.23%, range 0–3%. *CAT-SKL-EP* was usually undetectable in the eluates. There are about 44000 mRNAs per procyclic trypanosome [18,19]. If we assume that the result for *TUB* mRNA is representative of all other contaminating mRNAs, then for every 25 molecules of *3SBP-CAT-SKL* mRNA in the preparation (0.063 x 400) there are 100 contaminating mRNAs (0.0023 x 44000). Thus although we achieved 22-fold purification of the reporter mRNA, 80% of the mRNA in the preparation was *not* our reporter.

We also assessed protein purification by scanning four gels stained with Coomassie and silver. The pooled polysomes had, on average, 13-fold less protein than the cleared lysate. The amount of protein eluted from the streptavidin column was 1100-1400-fold less than that in the lysate, whether or not the starting lysate included 3SBP-tagged polysomes. This result is consistent with our estimate that most of the mRNPs in the preparations were not our reporter.

Four independent purifications were sent for mass spectrometry. In each case we compared preparations made with cells expressing the reporter with and without the SBPs. 660 different proteins were found (threshold of 2 peptides) in at least one of the *3SBP-CAT-EP* samples. These included 32 ribosomal proteins, and numerous other abundant proteins such as cytoskeleton and chaperones, as well as metabolic enzymes (S1 Table). About half of the proteins had also been detected in our older mass spectrometric survey of polysomal proteins [21]. Whereas we had previously found two eIF4E homologues, eIF4E3 and eIF4E4, in polysomes [21], the *3SBP-CAT* and the control preparations contained eIF4E4 but not eIF4E3. To look for significant enrichment of proteins in the *3SBP-CAT* preparation, we selected proteins that had been detected in three out of the four *3-SBP-CAT* samples, then compared the four replicate results with and without the 3SBPs. Using a threshold of at least 2-fold enrichment and Student t-test value of p<0.05 (either paired or unpaired), no protein was significantly enriched in the 3-SBP-CAT sample. Of the 23 detected proteins with RNA binding domains, two were at least 2-fold enriched in two of the four experiments: they were a protein with histone RNA binding domains encoded by Tb927.3.1910, and ZC3H13.

Developmentally regulated expression of *EP* procyclin depends on a U-rich region in the 3'-UTR [17,22]. Three proteins that are known to bind to U-rich regions—DRBD3 [23,24], UBP1/UBP2, and RBP3 [25–27]—were detected in all preparations to similar extents. U-rich regions are common in trypanosome 3'-UTRs, and it is quite possible that in procyclic forms, the *EP* 3'-UTR is associated mainly with abundant, relatively non-specific RNA-binding proteins. Other RBPs represented by at least 2 peptides in at least 6 of the 8 tested preparations, including the negative control, were ZC3H22, RBP42, DRBD18, DRBD11, PUF1, PUF6, and the protein encoded by Tb927.11.14220.

### Association of ZC3H11 with the *HSP70* 3'UTR

Our work with the *3SBP-CATSKL-EP* reporter had shown that our method was suitable for purification of a polysomal reporter mRNA, but had provided no evidence that we could show co-purification of an RNA-binding protein that was specifically associated with that mRNA. In order to do this, we needed to investigate a well-characterised, specific, RBP-mRNA interaction. For this, we chose the CCCH-domain protein ZC3H11. ZC3H11 binds to a (UAU)_7_ sequence in the 3'-UTR of *HSP70* mRNA [28]. ZC3H11 can interact with itself, and several ZC3H11 molecules are likely to bind to each *HSP70* mRNA, in a complex which includes other proteins, including MKT1 and PBP1 [29].

To test our method for the ability to co-purify a bound protein, we therefore used a *3SBP-CATSKL* mRNA containing the *HSP70* 3'-UTR (Fig 3A). The *HSP70* mRNA is relatively stable [20], so is suitable for abundant expression. As a negative control, we used the same reporter mRNA without the (AUU) repeat (Fig 3A). We expected both reporters to purify on the streptavidin matrix; the only difference should be whether or not ZC3H11 was also purified. The reporter expression levels were similar to those for *CAT-EP* (not shown). Thus again the reporter levels are similar to those of the *HSP70* mRNA, which has roughly 100 copies per normal procyclic cell [19] and about 400 after a 39°C heat shock (I. Minia and C. Clayton, ZMBH, manuscript in preparation). We expressed our reporters in trypanosomes expressing ZC3H11 with an N-terminal V5 tag from the endogenous locus [28] (i.e. not over-expressed). ZC3H11 is detectable only after heat shock. Before cell extract preparation, the trypanosomes were therefore heat-shocked for 1h at 39°C.

We purified the polysomal reporters as before, but this time we monitored not only the target mRNAs, but also associated proteins. Endogenous *HSP70* mRNA now served as a negative control for the mRNA purification since *HSP70* mRNA remains on the polysomes after heat shock [20]. At the mRNA level, purification of the two new reporters worked as well as, or better than, for the *3SBP-CATSKL-EP* mRNP (Figs 2C and 3B). The protein results showed that ribosomal protein S9 was present in similar amounts in both eluates (Fig 3C). The abundant cytosolic protein trypanothione reductase (TR) was also present in both, at a lower level (Fig 3C). In contrast, ZC3H11 was reproducibly enriched in the *3SBP-CATSKL-HSP70* mRNP relative to the *3SBP-CATSKL-HSP70delAUU* mRNP (Fig 3C and 3D). This showed that our method could purify a bound RNA-binding protein in a sequence-specific manner.

Interestingly, the specific purification of ZC3H11 was obtained only if the cells were UV-irradiated before lysis. In theory, cross-linking to RNA ought to change the mobility of the ZC3H11 complex in the polyacrylamide gel, but we could see no evidence for this, since incubation of the beads with RNase prior to boiling did not increase ZC3H11 detection. It is, however, known that UV cross-linking has a rather low efficiency [30]. Since ZC3H11 is part of a complex, cross-linking of one ZC3H11 to the mRNA may be sufficient to stabilise the interactions of additional bound—but not-cross-linked—ZC3H11 molecules. (Since UV-cross-linking reproducibly reduced the height of the polysomal peak—perhaps because of stress—we did not use it for the *3SBP-CAT-EP* experiments.)

Treatment of the matrix-bound polysomes with RNase ought to release proteins that are associated with the matrix only via intervening mRNA. This method has been employed successfully for mRNPs that are purified via aptamers in the mRNA sequence [8]. In one trial experiment, after binding of *3SBP-CAT-HSP70* polysomes to the streptavidin beads, we treated them with RNase using the published conditions then looked for elution of ZC3H11. As a positive control, we then boiled the beads as before. Unfortunately no detectable ZC3H11 was eluted using RNase (not shown). We do not understand this result. Although the initial binding reaction contains RNase inhibitor, extremely little should remain after the extensive wash steps. It is possible that further adjustments to the procedure would allow RNase-mediated protein elution.

We next looked to see whether we could see any specific association of MKT1 with the *3SBP-CATSKL-HSP70* mRNP. We could not see any specific association (Fig 3E). MKT1 can associate with various different RNA-binding proteins [29], and preliminary results (M. Terrao, unpublished) also suggest that it is bound to many mRNAs. If this is the case, MKT1 may be associated with several of the non-SBP mRNAs that contaminate our preparation.

The Western blot result for ZC3H11 suggested that it should be detectable by mass spectrometry. To find out whether this was the case, we subjected three *3SBP-CATSKL-HSP70* mRNP preparations to mass spectrometry, looking not just for total protein composition, but also specifically for modified or unmodified peptides from ZC3H11. In total, 1020 different proteins were detected (S1 Table). Unfortunately however, only one modified peptide of ZC3H11 was detected in just one of the preparations, and even this was below the standard threshold. ZC3H11 is intrinsically difficult to detect by mass spectrometry due to heavy phosphorylation ([28]; I. Minia and C. Clayton, ZMBH, manuscript submitted). We also failed to find the V5 tag, which should remain joined to the serine-rich N-terminus after trypsinisation. The specific aim of this part of the study was to find out whether mass spectrometry could detect significantly more ZC3H11 in the *3SBP-CATSKL-HSP70* mRNP than in the *3SBP-CATSKL-HSP70delAUU* mRNP. Since we could not detect ZC3H11 in the *3SBP-CATSKL-HSP70* positive control we decided not to proceed with mass spectrometry of the *3SBP-CATSKL-HSP70delAUU* negative control.

## Discussion

In this paper, we describe a method that can purify at least 8% of a reporter mRNA from polysomes, giving a preparation in which the reporter mRNP is 25-fold enriched and constitutes about 10% of mRNPs. A few simple calculations show that the level of purification and yield would suffice to detect a specific trypanosome protein-RNA interaction only under very particular circumstances:

1. Will a specifically bound protein be detectable? A procyclic trypanosome contains about 4x10^8^ protein molecules. If the cells contain 400 copies of the reporter, and a single copy of an RNA-binding protein is attached to each, the initial molar ratio of the specific target RNP to total protein is 1 in a million. After 1200-fold purification (at the protein level), with 10% yield of the specific mRNP, the molar ratio of specific mRNP to total protein is approximately 1:2000. If two proteins are bound per mRNP, the ratio becomes 1:1000. Quantitative mass spectrometry procedures that involve mixing of the positive sample with the negative control result in reduced sensitivity, since the amount of any protein that is present only in one preparation is halved. However, the numbers suggested here should be within reach of sensitive state-of-the-art mass spectrometers. If the mRNA is less abundant—or the protein can dissociate during the procedure—the prospects are bleak.
2. How selectively must a protein bind in order to be detected as specific to a particular mRNP? Usually, about 80% of the mRNAs in the purified preparation are contaminants. Suppose we set a threshold that for a protein to be considered specific, it must be three-fold more abundant in the purified preparation than in the negative control. In all cases examined so far, regulatory RNA-binding proteins have been found to bind to a cohort of different, but often co-regulated, transcripts. All of the untagged mRNPs that contain a particular RNA-binding protein will be present in both the experimental and the control fractions. If there are too many of these, it will not be possible to detect specific binding of the protein to the reporter. For ZC3H11, specificity was seen because the proportion of mRNAs that it is bound by ZC3H11 is relatively small. Eighty-seven different mRNAs were at least 2-fold enriched in a ZC3H11 pull-down [28] and there are 1700 of these mRNAs in procyclic forms after a 39°C heat shock (I. Minia and C. Clayton, ZMBH, manuscript in preparation). In the purified *3SBP-CATSKL-HSP70* mRNP preparation there should be 25 molecules of purified *3SBP-CATSKL-HSP70* reporter mRNA (6.3% of 400 per cell), for every 4 other mRNAs that can bind ZC3H11 (0.23% of 1700)—ratio of 6:1. Detection of specific binding of any protein therefore depends critically on the total number of mRNA targets, and the number of molecules of that protein bound to each target mRNA. In general, the method should have the capability to detect any protein that binds to less than about 8% of all mRNAs.

From the above considerations, it is evident that it ought to be possible to detect some proteins that are specifically associated with an mRNA using 3-SBP-nascent-peptide purification. Moreover, the method is still open to improvement. Formaldehyde treatment would be an attractive way to reversibly stabilise the mRNPs, more efficiently than UV-cross-linking. Use of 4-thio-uracil/uridine might improve the cross-linking efficiency but it is not taken up efficiently by trypanosomes (our unpublished results and [31]). Since formaldehyde-treated trypanosomes cannot be lysed with detergent, and we feared that sonication would break the polysomes, we tested formaldehyde treatment of the cell lysate. Sucrose gradients of such preparations, however, did not give recognisable polysome profiles, presumably because of excessive cross-linking. Background from organellar proteins could be reduced by non-detergent-based breakage followed by centrifugation. Higher expression of the 3SBP-reporter would improve both the yield and purity, but it is risky because non-physiological levels of the mRNA might have abnormal interactions. It is possible that better purification could be obtained using magnetic beads, using different binding and wash conditions from those we tested. Indeed, since we found it so difficult to elute the SBP-bound polysomes, more stringent wash conditions are another option: the major restriction would be that the streptavidin must not be denatured. Greater specificity might also be obtained by elution using RNAse: although we failed to achieve this, we only tried one set of conditions.

Two publications [10,11] recently described extremely efficient purifications of the Xist RNA, together with its bound proteins, from mammalian cells. Both methods involve cross-linking (with UV or formaldehyde) and fragmentation of the RNA prior to purification using oligonucleotides that target the entire length of the 17kb mouse Xist mRNA. Yields were 60–70% [10,11] with 5000-fold enrichment [11]. The binding stoichiometries of the identified proteins, relative to Xist, are not yet known, so binding at multiple sites along Xist could have facilitated their identification. The only other limitation is that only proteins that are directly bound to the RNA can be obtained. Such approaches will probably always be restricted to RNAs that are reasonably abundant. Nevertheless it is clearly possible that these methods could be adapted for mRNAs.

In conclusion: The method described here gives higher yields and purity than other previously-described methods that were designed to isolate complete mRNPs, including not only the proteins that are directly bound to mRNA, but also those that interact with them. Our method could also be combined with a second one that relies on direct selection of the RNA sequence. To do this, a simple calculation suggests that starting material from at least 10^11^ cells would be required (S2 Text). This would be logistically challenging, but not impossible.

## Materials and Methods

### Plasmid construction and trypanosomes

Plasmids were constructed by a combination of restriction digestion and PCR. All are designed to integrated into an rRNA spacer region, and contain an inducible *GPEET* promoter and a hygromycin resistance selectable marker. The reporter mRNA has an *EP* 5'-UTR. Plasmids were as follows (reporter protein coding region and 3'-UTR): pHD2306: 3SBP-CAT-SKL, *EP* 3'UTR; pHD2319: CAT-SKL, *EP* 3'UTR; pHD2392: 3SBP-CAT-SKL, *HSP70* 3'UTR; pHD2415: 3SBP-CAT-SKL and *HSP70delAUU* 3'UTR. Plasmids were linearized with *Not* I, and transfected into *T*. *brucei* 427 procyclic forms expressing the *tet* repressor (pHD1313), which were grown and transfected as previously described ([21] and see S1 Text)

### Polysome purification

The detailed protocol is provided in S1 Text and S1 Fig. For a typical proteomic experiment we prepared six 4 ml gradients, and loaded up to 3x10^8^ cells per gradient, but we used 6x10^8^ after heat shock. Briefly, cells (maximum density 2 x 10^6^/ml) were concentrated by centrifugation, resuspended in medium without serum then UV cross-linked on ice (0.3 J/cm) in a Stratalinker [32]. Cycloheximide was added to 100μg/ml. The cells were left at room temperature for 5 min, pelleted, and resuspended in polysome buffer (20mM Hepes pH 7.5, 25mM NaCl and 5mM MgCl_2_. [1], 5 μg/ml leupeptin, 0.5 mg/ml heparin, 2mM DTT, 100 μg/ml cycloheximide, 40U/μl rRNasin, 200mM Sucrose, 1pill/10mL of EDTA-free Protease Inhibitors). NP40 (final 02%) was added and the cells were lysed by passing 15–30 times through a 21G needle. Lysis was monitored microscopically after diluting the suspension 100-fold in polysome buffer without detergent. Extracts were cleared by centrifugation and the supernatants were loaded onto 15–50% sucrose gradients [20]. Meanwhile, streptavidin beads were blocked with 0.1 mg/ml tRNA and 0.4 mg/ml heparin, in 20mM Hepes (pH 7.5), 25mM NaCl and 5mM MgCl_2_ [1]. Collected polysomes were added to the beads (25μl for 400μl of polysome suspension, or 150 ul beads for polysomes from 1x10^9^ cells), and tumbled for an hour at 4°C. After mild centrifugation, the beads were washed three times with the same buffer. Additional washes did not improve purity.

### RNA and protein analysis

To extract RNA samples for Northern blot, beads were treated with Proteinase K for 20 min at 42°C, and RNA was then extracted using Trizol, separated on formamide-formaldehyde gels, blotted onto Nytran, cross-linked, and hybridized with ^32^P-labelled probes. The signal was detected using a phosphorimager and quantitated using ImageJ. Proteins were extracted from the beads by direct boiling in SDS-PAGE sample buffer, and analysed by Western blotting using antibodies to the V5 tag (AbD serotec), ribosomal protein S9, and trypanothione reductase (kind gift from L. Krauth-Siegel, Biochemiezentrum Heidelberg) with detection by ECL.

Mass spectrometry was done using an LTQ Orbitrap Velos (1) at the University of Dundee, Scotland. Gel bands re-suspended in 50ul (1% formic acid) and 15ul injected onto a EasySpray column (75um x 50cm) and run over a 65 min linear gradient (2–40%B).

## Supporting Information

S1 Fig Supplementary Figure S1: Steps in the purification procedure. (PDF)

S1 Table Mass spectrometry results for *CAT-EP*, *3SBP-CAT-EP* and *3SBP-CAT-HSP70*. Peptide counts for each identified protein are listed. Note that since many more peptides were identified in the *3SBP-CAT-HSP70* experiments than with the other baits, the peptide counts from this reporter cannot be compared with those from the others. The protein annotations are from TriTrypDB (http://tritrypdb.org/tritrypdb/), with manual alterations.
(XLS)

S1 Text Supplementary document S1: Detailed method description. In the flow chart, steps of the purification are grouped by day and shown in boxes. MS, mass spectrometry.
(PDF)

S2 Text Supplementary document S2: Summary of the various variations that were tested, and some additional possibilities. (PDF)
