# Supplementary material for: Purification of Messenger Ribonucleoprotein Particles via a Tagged Nascent Polypeptide
Source: PLoS One. 2016 Jan 25;11(1):e0148131. doi: 10.1371/journal.pone.0148131 (PMC4726818; doi:10.1371/journal.pone.0148131)
Supplement: S1 Fig — (PDF) [file pone.0148131.s001.pdf]

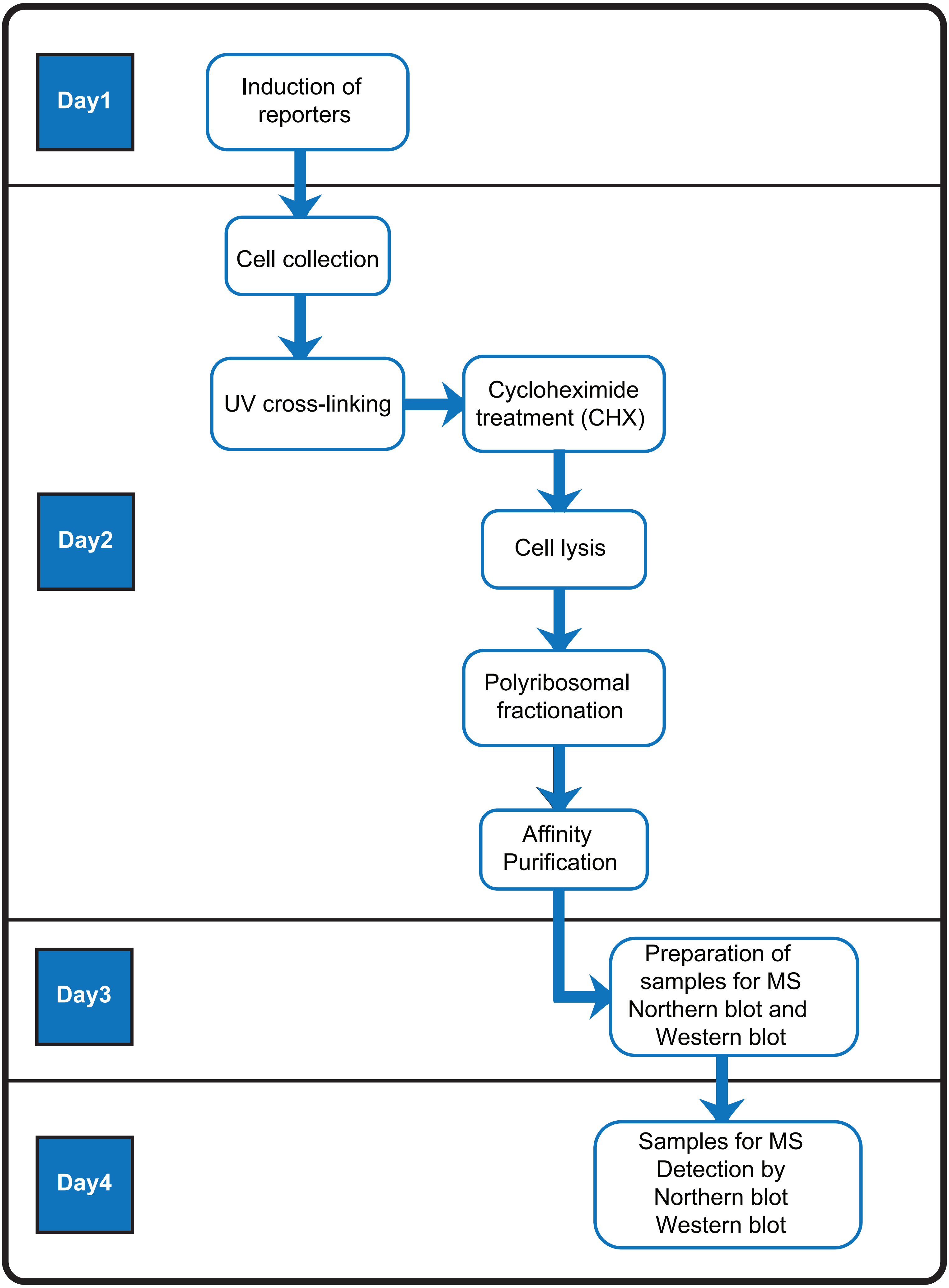
Day1
Induction of reporters
Cell collection
UV cross-linking
Cycloheximide treatment (CHX)
Day2
Cell lysis
Polyribosomal fractionation
Affinity Purification
Day3
Preparation of samples for MS Northern blot and Western blot
Day4
Samples for MS Detection by Northern blot Western blot
