# Supplementary material for: Purification of Messenger Ribonucleoprotein Particles via a Tagged Nascent Polypeptide
Source: PLoS One. 2016 Jan 25;11(1):e0148131. doi: 10.1371/journal.pone.0148131 (PMC4726818; doi:10.1371/journal.pone.0148131)
Supplement: S1 Text — In the flow chart, steps of the purification are grouped by day and shown in boxes. MS, mass spectrometry. (PDF) [file pone.0148131.s003.pdf]

DETAILED PROTOCOL

Materials

Reagents

Reagents needed for procyclic cell culture

- MEM-PROS mixture (Biochrom, cat. no. T031-01)
- MEM vitamins (SIGMA, cat. no. 58970C)
- MEM non escential aminoacid solution (SIGMA, cat. no. M7145)
- Phenol red (SIGMA, cat. no P3532)
- FCS (Biochrom; cat. no. S0615)
- Hemin (SIGMA, cat. no H9039)
- Penicillin/Streptomycin (SIGMA, cat. no. P0906)

Reagents used in the polysome and affinity purification buffers

- Tris pH 7.5 (ROTH, cat. no. 4855.2)
- Potassium chloride (MERK, cat. no. 1.04936)
- Magnesium chloride hexahydrate p.A. (AppliChem, cat. no. A3618.2050)
- Leupeptin (SERVA, cat. no. 51867.02)
- Heparin sodium (SERVA, cat. no. 24590.02) ! CAUTION irritant (eyes, skin and respiratory). Avoid contact with skin by the use of gloves.
- DTT, dithiothreitol (SERVA, cat. no. 39759) ! CAUTION acute toxicity, skin irritation, eye irritation and specific target organ toxicity. Use eyeshield, faceshield, dust mask and gloves.
- IGEPAL CA-630, Nonident P-40 (NP-40; SIGMA, cat. no. I3021)
- Cycloheximide TLC (SIGMA, cat. no. C7698-1G) ! CAUTION danger, acute toxicity (oral, dermal and by inhalation). Germ cell mutagenicity, carcinogenic, also reproductive toxicity, specific target organ toxicity and hazardous to the aquatic environment (acute and chronic). Use eyeshield, faceshield, filter mask and gloves.
- rRNasin Promega
- tRNA from *Escherichia coli* MRE600 (ROCHE, cat. no. 10109550001)
- Protease Inhibitors complete tablets, Mini, EDTA-free (ROCHE cat. no. 04693159001)

Reagents used in the sucrose gradients and fractionation

- D(+)-Saccharose (ROTH, cat. no. 4621.1)
- Silicone paste Baysilone (GE Bayer, cat. no. HOCHVISKOS)

Reagents necessary for RNA extraction and Northern blot

- EDTA disodium salt dehydrate p. A. (AppliChem, cat. no. A3553)
- SDS, dodecylsulfate sodium salt in pellets (SERVA, cat. no. 20765.01)
- Proteinase K (MERCK, cat. no. 1.24568.0100)
- peqGOLD TriFast^{TM}FL for RNA extraction of liquid samples (Peqlab, cat. no. 30-2120) and peqGOLD TriFastTM for RNA extraction of pellets (Peqlab, cat. no. 30-2010) ! CAUTION it contains phenol and guanidinium thiocyanate. Toxic in contact with skin (causes burns) and if swallowed. Use gloves and eyeshield, work must be done under a fume hood.
- Chloroform (SIGMA, cat. no. 32211) ! CAUTION skin and eye irritation, acute toxicity (oral, dermal and by inhalation). Germ cell mutagenicity, carcinogenic, also reproductive toxicity and specific target organ toxicity. Use gloves and eyeshield, work must be done under a fume hood.

- 2-propanol (AppliChem, cat. no. A3267)
- 100% Ethanol (SIGMA, cat. no. 32205)
- 70% ethanol (diluted from 100%)
- Glycogen (SIGMA, cat. no. G8751)
- UltraPureTM Agarose (Life Technologies, cat. no. 16500)
- Formaldehyde solution 37%, p.a., ACS (ROTH, cat. no. 4979.1) ! CAUTION danger, acute toxicity (oral, dermal and by inhalation). Germ cell mutagenicity, carcinogenic, also reproductive toxicity, specific target organ toxicity and hazardous to the aquatic environment (acute and chronic). Also corrosive to metals, skin corrosion and serious eye damage. Use eyeshield, faceshield, filter mask and gloves, work must be done under a fume hood.
- 100% Formamide, deionized (Ambion, cat. no. AM9342) ! CAUTION
  Harmful by inhalation, in contact with skin or if swallowed; it also causes burns. Formamide should be used with protective gloves, glasses and clothing.
- MOPS (SERVA, cat. no. 29836.02)
- MOPS buffer (see REAGENT SETUP)
- Tri-Sodium citrate dehydrate SSC p. A. (AppliChem, cat. no. A2403)
- Methylene blue (SERVA, cat. no. 29198) for methylene blue in solution (see REAGENT SETUP)
- Salmon spermidin, (see REAGENT SETUP) deoxyribonucleic acid, low molecular weight (SIGMA, cat. no. 31149)
- 50X Denhardt's solution (see REAGENT SETUP)
- 32P alpha dCTP Hartman Analytics SRP 205 ! CAUTION
- Nucleotide removal kit
- Pre hybridization buffer (see REAGENT SETUP)
- Hybridization buffer (see REAGENT SETUP)
- Wash buffer #1 (see REAGENT SETUP)
- Wash buffer #2 (see REAGENT SETUP)

Reagents necessary for affinity purification

- Streptavidin sepharose high performance beads (GE Healthcare, cat. no. 17-511301)
- Polysome buffer (see REAGENT SETUP)
- Blocking buffer (see REAGENT SETUP)
- Affinity purification buffer (see REAGENT SETUP)

Cells

- Cells: procyclic trypanosomes (see REAGENT SETUP)
- Zimmerman's Post Fusion Medium (ZPFM) for transfection (see REAGENT SETUP)
- Tetracyclin added to a final concentration of 200 ng/ml (SIGMA, cat. no. T3258)

Reagents used for Western blot and Coomassie

- Running buffer 20X NuPAGE MOPS SDS (Life Technologies, cat. no. NP0001)
- TBE Running buffer 5X (NOVEX by Life Technologies, cat. no. LC6675)
- Sample buffer (4X) NuPAGE LDS (Life Technologies, cat. no. NP0008)
- Fixing solution (see REAGENT SETUP)
- Acetic acid 100% (AnalR NORMAPUR, cat. no. 20104.298)
- 40% ethanol (diluted from 100%)
- Colloidal Coomassie (see REAGENT SETUP)
- Transfer buffer (see REAGENT SETUP)
- Milk powder blotting grade, fettarm (ROTH, cat. no. T145.2)
- Western lightning Plus-ECL (Perkin Elmer, Inc., cat. no. NEL104001EA) and for more sensitivity the Western lightning Ultra (Perkin Elmer, Inc., cat. no. NEL112001EA)

- Antibodies used:
    - Mouse Anti V5-TAG (AbD serotec, cat. no. MCA1360)
    - ECL^{TM}Anti-Rat IgG, Horseradish Peroxidase linked whole antibody (GE Healthcare, cat. no. NA935V)
    - ECL^{TM}Anti-Rabbit IgG, Horseradish Peroxidase linked whole antibody (GE Healthcare, cat. no. NA934V)
    - ECLTMAnti-Mouse IgG, Horseradish Peroxidase linked whole antibody (GE Healthcare, cat. no. NA931V)

Equipment

- Electroporation system, BTX ECM600 Electro Cell Manipulator (Artisan Technology group, http://www.artisantg.com/)
- Cuvettes for electroporation 25 x 2mm gap (Peqlab, cat. no. 71-2020)
- Petri dishes, 145 x 20mm radius (Greiner bio-one, cat. no. HSO451)
- Protein LoBind tube 1.5ml (Eppendorf, cat. no. 022431081)
- DNA LoBind tube 2.0ml (Eppendorf, cat. no. 022431048)
- Centrifuge tubes for sucrose gradients, 7/16x2-3/8 P.A. tube (BECKMAN COULTER, cat. no. 328874)
- Ultracentrifuge SORVALL Discovery 90SE (Thermo Scientific, http://www.fishersci.com.sg/)
- Ultracentrifuge rotor SW60Ti, swinging buckets 11 x 60 mm (BECKMAN COULTER, cat. no. 41103909)
- Density gradient former fractionator ISCO160. This system uses the retriever Foxy Jr Fraction Collector to collect separated fractions. Contains a tube holder and UA-6 UV/VIS detector with 454 and 280nm filters. The machine is able to produce a continuous absorbance profile as the gradient is displaced from the bottom up by a dense chase solution (60% sucrose) and collected in precise measured fractions. (TELEDYNE ISCO. www.isco.com)
- Northern blot membrane, Amersham Hybond^{TM}-N^{+} (GE Healthcare, cat. no. RPN303B)
- Western blot transfer membrane from cellulose nitrate, 0.45 µm 300 x 3000 mm (Neolab, cat. no. 711130641BL)
- Pre-cast protein gels NuPAGE 4-12% Bis –Tris Gel (NOVEX by Life Technologies, cat. no. NP0335PK2)
- Chamber to run the NuPAGE gels, XCell II^{TM} Mini Cell (NOVEX by Life Technologies, cat. no. EI9001)
- Heating block at 42°C necessary for proteinase K treatment of the RNA.
- Heating block at 95°C for Western blot samples and to prepare the radioactive probe.
- Heating block at 65°C necessary to denature the RNA for Northern blot.
- Stratalinker^{R} UV Crosslinker 2400 for 230V (Stratagene, cat. no. 400076)
- ImageQuant LAS 4000 to develope Western blot (GE Healthcare, cat. no. 28-9558-10)
- Phosphorimager FLA 7000 to develop Nothern blots (GE Healthcare, http://www.gelifesciences.com/)
- Imaging Cassettes BAS 2040 and films (Fujifilm, http://www.fujifilm.com/)
- Laminar flow hoods to work with procyclic trypanosomes in the S1 cell culture and to extract RNA and perform Northern blots.

Reagent setup

Media for procyclic cells MEM-PROS mixture was mixed with MEM vitamins, MEM non-essential amino acid solution and 100mg phenol red (pH 7.4). The media was sterilized by filtration and stored at 4°C. One night before use the media was supplemented with heat-inactivated FBS 10%(v/v), 7.5 mg/l hemin and 50U/ml penicillin/streptomycin.

Cells Monomorphic Lister 427 procyclic trypanosomes were cultured in supplemented MEM-Pros medium at 27°C to a density of 1 x 10^6 cells/ml. All work was done under sterile conditions in a S1 laboratory in a laminar flow hood.

Zimmerman's Post Fusion Medium (ZPFM) 132mM NaCl, 8mM KCl, 8mM Na_2HPO_4,1.5mM KH_2PO_4,1.5mM MgAc x 4 H_2O, 90 µM $Ca(OAc)_2$; pH 7.0, filter-sterilized and stored at 4°C

Generation of stable cell lines in trypanosomes A total of $1x10^7$ cells were used per transfection. The cells were washed once with PBS and resuspended in 0.5 ml of ZPFM. Then, approximately 8 µg of digested plasmid was mixed with the cells and transferred to a 2mm gap cuvette for electroporation. The settings used were 1.5 kV and resistance R2 in the BTX electroporator. On the next day, the selective antibiotic was added and the cells plated in serial dilution on a 24 well plate. Proliferating clones were picked later and checked for expression.

Polysome buffer 20mM Hepes (pH 7.5), 25mM NaCl and 5mM $MgCl_2$. (Schutz et al 1977). At the moment of the experiment add: 5 µg/ml leupeptin, 0.5 mg/ml heparin, 2mM DTT, 100 µg/ml cycloheximide, 40U/µl rRNasin, 200mM Sucrose, 1pill/10mL of Protease Inhibitors EDTA-free and 0.2% NP40 (the detergent was only added at the moment of lysis).

Blocking buffer 20mM Hepes (pH 7.5), 25mM NaCl and 5mM $MgCl_2$ (Schutz et al 1977). At the moment of the experiment 0.1 mg/ml tRNA and 0.4 mg/ml heparin were added.

Affinity Purification buffer 20mM Hepes (pH 7.5), 280mM NaCl, 5mM $MgCl_2$. At the moment of the experiment add 0.4 mg/ml of heparin.

Proteinase K buffer 0.2% SDS, 8mM EDTA and 0.8 µg of Proteinase K. Incubate 20 min at 42°C

Sucrose gradients Gradients were prepared by adding the AP buffer supplemented with 5 µg/ml leupeptin, 0.5 mg/ml heparin and 2mM DTT to 15% and 50% sucrose mixtures. The sucrose layers were made manually, by loading first the 15% sucrose into the centrifuge tubes and later the 50% sucrose solution was loaded at the bottom of the tube, pushing the 15% solution up. The upper part of the centrifuge tubes were covered with parafilm and left in a 90° position for 2 h. Gradients were stored at -80°C.

Equipment setup

Sucrose density gradient fractionation The ultracentrifuge was pre-cooled before the centrifugation by starting the vacuum. The SW60 rotor and tubes were kept at 4°C overnight. 15-50% sucrose gradients were left to thaw overnight at 4°C in a place without external movement. ! CRITICAL STEP.

Once the gradients were thaw, the clear lysate was loaded on top of the gradients at 4°C, without disturbing the sucrose layers and without making bubbles while loading. Also there must be 1-2 mm space left after loading in order to avoid the implosion of the tubes while ultracentrifugation ! CRITICAL STEP. The gradients were ultra centrifuged at 164,326 *g* for 2 h in the SW60 rotor.

Fractionator setup The fractionator was assembled as indicated by the manufacturers' instructions (TELEDYNE ISCO. www.isco.com) and the tubing cleaned once with distilled

water. Before the fractionation started, 60% sucrose was pumped into the tubes taking care that there were no bubbles left in the tubing. The needle and the equipment were adjusted properly to avoid leakage of the gradients and the UV-lamp turned on approximately 15 min in advance before its use.

The polyribosome profile was recorded at 454 nm and takes approximately 12 min per gradient. For large-scale experiments, 6 gradients (a full rotor) were used. The profile can be recorded in paper or in the computer.

At the end of the fractionation, the fractionator was washed twice with water and once with ethanol 70%; in order to avoid the formation of fungus or bacteria in the tubing, which can later contaminate the sample.

Blocking step The streptavidin beads were blocked with 1ml of blocking buffer (see REAGENT SETUP) per 25 µl beads before the affinity purification. 25 µl is suitable for polysomes from 7 x 10^8 cells - see File S2. The beads were tumbled within the blocking buffer for 1 hour at 4°C, spun down at 400 *g* for 2 min and then washed twice with polysome buffer and once with affinity purification buffer (see REAGENT SETUP). Before the incubation with the polysomal fractions all buffer was removed from the beads.

Procedure

Induction of the 3SBPs-CAT mRNA TIMING Day1, 5 min

1. Add 200 ng/ml tetracycline to the culture in order to induce the expression of the reporters and leave to grow overnight. Depending on the expression of the vector the induction time can be reduced.

Collection of cells and cycloheximide treatment TIMING Day2, 30 min

2. Cells ($3x10^8$ procyclic trypanosomes per gradient) are collected by centrifugation at 500 *g* for 10 min at 4°C. (After heat shock double the number can be used because there are fewer polysomes.)

From this step onwards everything must be done on ice or at 4°C if possible ! CRITICAL STEP.

3. Wash the pellet with 10 ml of media without serum per $8x10^6$ cells and distributed in a Petri dish (145mm radius).
4. Cross-link once the RNA and protein complexes at 254nm (0.3 J/cm) using the UV StratalinkerTM 2400
5. Collect the cells in a 50ml falcon, add 100 µg/ml of cycloheximide and incubate for 5 min at room temperature.
6. Spin down the cells for 8 min at 500 *g*, resuspend the pellet in 1ml of polysome buffer without detergent and transferred to a 1.5ml LoBind eppendorf tube.
7. Spin the cells down for 3 min. In this step the cell pellets could be frozen in liquid nitrogen and store at -80°C.

Cell lysis TIMING Day2, 2 h + 20 min

8. Resuspend the cells in 200µl of polysome buffer with detergent.
9. Lyse by passing the cells 15 to 30 times through a 21G 11/2 needle (? TROUBLESHOOTING).
   CRITICAL STEP The lysis must be done at 4°C and can be supplemented with RNase inhibitors. Also adjust the salt concentration to 120 mM KCl.
10. Clear the lysate by centrifugation at 16,000 *g* for 10 min

11. Load the clear lysate carefully in the sucrose gradients and ultracentrifuge the gradients at 164,326 *g* for 2 h (? TROUBLESHOOTING).
While the centrifuge is running clean and assemble the fractionator. Also wash the blocking buffer from the streptavidin beads (see EXPERIMENTAL SETUP).

Polyribosomal fractionation TIMING Day2, 20 min

12. Fractionate the samples by time (22 seconds each fraction) using the ISCO160 fractionator; this will give 400 µl fractions approximately. The lysate of 6-8x10^8 cells can be loaded into the sucrose gradients without saturation. Each gradient takes approximately 12 min to collect and detect (? TROUBLESHOOTING).
CRITICAL STEP Make sure that the needle is assemble properly and that the gradient is tightly fixed to the fractionator in order to avoid the leakage of the sample.
13. Pool the polyribosomal fractions and take samples for Northern blot and/or Western blot (? TROUBLESHOOTING) If the fractionator is not in the cold room one can pre cool the tubes (at -20°C) and as soon as the sample is collected put it on ice.

Affinity Purification TIMING Day2, 2h

14. Add the polyribosomal fractions to the pre-blocked Streptavidin sepharose beads and tumble for 1 h. Perform all steps on ice or at 4°C ! CRITICAL STEP
15. Spin down the beads at 900 *g* for 3 min and leave on ice until the beads settle. Due to the high concentration of sucrose, this step can take approximately 20 min.
16. Take a sample of the unbound fraction for Northern and/or Western blot and wash the beads 3 times with affinity purification buffer.
17. Take samples of the wash for Northern and Western blot (? TROUBLESHOOTING)

Preparation of samples for Mass spectrometry Day3, 30 min + overnight staining

18. Add 12.5µl of 4x sample buffer and boil the beads at 95°C for 10 min.
19. Load the samples in a pre-cast protein gels NuPAGE 4-12% Bis –Tris Gel (? TROUBLESHOOTING) and run the samples for 3 cm.
20. Stain with Coomassie brilliant blue and wash overnight.

Mass spectrometry, Day4, 5 min

21. Cut the samples as desired and send the bands to mass spectrometry (? TROUBLESHOOTING).

TIMING

Step 1 (day 1) approximately 5 min
Steps 2-17 (day 2) approximately 5 h and 10 min
Steps 18-20 (day 3) approximately 30 min and overnight incubation
Step 21 (day 4) approximately 5 min
See Figure 2

Troubleshooting

Table1 Troubleshooting table

Problem	Possible reasons	Suggestions
Cell lysis		
Not enough polyribosomes	Incomplete cell lysis	Use a 21G 11/2 needle for

obtained		the lysis, smaller needles will take more time for lysis and RNA degradation might occur.<br>Check the efficiency of lysis by dilution 1:100 in the buffer (before and after the lysis) and observe/count the cells in a Newbauer chamber (load 10µl of the dilution into the chamber)
Disturbed polysome profile	Overloaded sucrose gradient	Do not load more than $6x10^6$ cells per gradient. In case of heat shock treatment $8x10^6$ cells can be load
Higher background	Biotinylated proteins present in the lysate	Block the lysate using Avidin-agarose beads. Mix the lysate with 25µl of 50% Avidin slurry and tumble for 10 min at 4°C. Spin down the beads at 2400 *g* for 5 min and continue with the protocol in step 10
Cell fractionation		
Assembling the fractionator	Leakage of gradients	Fix the gradient to the fractionator using silicon paste and tightening the needle to the collector.
Disturbance of the gradients	Bubbles in the tubing	Pass 60% sucrose trough the tubing before starting the fractionation<br>Hit slightly the tubing to get rid of the bubbles while pumping 60% sucrose at full speed<br>Leave sucrose pass through the needle in order to ensure that there are not bubbles inside the needle<br>The 60% sucrose can be recover by pumping back and can remain at 4°C or even be frozen, if care is taken to avoid contamination.
Affinity purification		
SBP-tag not binding to the streptavidin beads	Not enough SBPs	At least 3 SBPs must be present in order to be able to

		purify the specific mRNP (Busby, M., et al., *Optimisation of a multivalent Strep tag for protein detection.* Biophys Chem, 2010. **152**(1-3): p. 170-7.)
	Not enough reporter mRNA produced	Calculate the number of mRNA in the cell. Compare *in vitro* transcription of the reporter with the in-vivo expression (see supplementary Figure S2)
Higher background	Beads used	Try different types of beads (magnetic beads other than sepharose)
	Beads not block	Use tRNA and heparin to block the beads before the affinity purification (see EQUIPMENT SETUP)
	Lysis method	Use of glass beads or an abrasive substance, such as, carbamide can reduce the background but can compromise the yield obtained. Total lysis with detergents can improve it.
RNA extraction-Norhtern blot		
RNA degraded	Presence of RNases in the sample	Before lysis add RNase inhibitors<br>Add RNase inhibitors while incubating with the streptavidin beads (step 14)<br>At the moment of fractionation, collect the samples in pre-cooled LoBind tubes<br>Perform the lysis and affinity purification steps at 4°C
Not enough RNA	Samples UV cross-linked, not good removal of proteins	Perform the Proteinase K treatment (see REAGENT SETUP) to remove covalently cross-linked proteins before RNA extraction
	Inappropriate TriFast reagent use	Use the TriFast^{TM}FL for liquid samples (samples from polysomes right after fractionation, such as input from polysomes, unbound

		fraction and wash). To extract RNA from the streptavidin beads use TriFast™
Western blot		
Samples difficult to load in the SDS-PAGE gel	High sucrose concentration	After boiling the beads (95°C for 10 min), place the samples in a 65°C heating block until loaded in the gel
Samples run not parallel in the SDS-PAGE	High salt concentration	Load at the side of the samples, 20µl of the Affinity Purification buffer (high salt buffer)
Mass spectrometry		
Few peptides identify by mass spectrometry	Dilution of the samples when using quantitative mass spectrometry	Not recommended to do quantitative MS when working with underrepresented RBPs
Higher number of unspecific peptides	Lysis method	Change the lysis method to carbamide but be aware that yield can be compromised<br>Use different controls for the analysis (unbound fractions to MS or compare with other purifications)
Not enough specific peptides detected	UV cross-linked samples	If known, perform the MS search using data of (modified or unmodified) peptides of the protein of interest
