# Supplementary material for: Purification of Messenger Ribonucleoprotein Particles via a Tagged Nascent Polypeptide
Source: PLoS One. 2016 Jan 25;11(1):e0148131. doi: 10.1371/journal.pone.0148131 (PMC4726818; doi:10.1371/journal.pone.0148131)
Supplement: S2 Text — (PDF) [file pone.0148131.s004.pdf]

Tested and possible adjustments in the protocol

Most tests were done at least twice.

Procedure	Pros / discussion	Cons
**Pre-lysis treatment**		
Cycloheximide	Stabilises the polysomes throughout the procedure	Build-up of ribosomes near start codon (not really a disadvantage)
UV cross-linking to achieve covalent binding of proteins to RNA	Bound proteins will wash off less easily. Complex stays stable after denaturation, so the eluate could be used for a second purification step that does not require intact protein-protein interactions.	Fewer high molecular-weight polysomes obtained. If cross-linking were very efficient, RNase treatment would be necessary to elute the proteins (not really a disadvantage).
Formaldehyde cross-linking to achieve covalent binding of proteins to RNA	More efficient cross-linking than UV; also will include important proteins that are not directly bound to RNA, part of mRNA-bound complexes.	If intact cells are fixed, they can be broken only by physical procedures (e.g. sonication) that break the mRNA. Treatment of lysates eliminated polysome profiles. Formaldehyde could cross-link too many molecules, reducing specificity.
**Cell lysis**		
Cytosolic extract, prepared by grinding with silicon carbide in isotonic (10% sucrose) buffer, centrifugation at 17,000 g for 1h.	None	Centrifugation at 17,000 g did not remove the 3SBP-CAT-SKL.
Cytosolic extract, prepared by grinding with silicon carbide in isotonic (10% sucrose) buffer, centrifugation at 33,000 g for 1h.	Mature 3SBP-CAT-SKL, glycosomal enzymes and cytoskeletal proteins are removed from the lysate. (80% of the CAT activity was in the pellet.) This not only removes contaminants but should also reduce the amount of streptavidin beads needed.	About 2/3 of the *CAT* RNA was lost in the centrifugation step. After loading on the polysome gradient, cytosolic lysate from at least 6×10^8 cells was needed in order to detect the *CAT* mRNA. Lysis with silicon carbide takes longer than NP40 (5-10 min to achieve 95% lysis, monitored microscopically). The extra centrifugation provides more possibility for RNA degradation. Overall too much polysomal RNA and protein is lost using this procedure.
Total (cleared) cell lysate made using NP40	More efficient polysome and RNA recovery. Purified *CAT* RNA readily detected from polysomes originating from 3×10^8 trypanosomes	Organellar and cytoskeletal proteins heavily contaminate the lysate that is loaded on the polysome gradient.

**Polysome gradient**		
Standard protocol according to ref 18; buffer with 120 mM KCl	Using cytosolic (glycosome-free) input lysates, the polysome purification removes 2/3 of the residual CAT activity.<br>Using NP40 lysates, the gradient gives10-fold purification at the protein level.<br>A different buffer with 280 mM NaCl gave slightly poorer resolution of the polysomes.	This step is time-consuming (2h centrifugation plus half-hour to collect 6 gradients). The maximum capacity of a 12-14 ml gradient would be about 1.2 x 10^9 cells, so no more than 8 x 10^9 cells could be used per centrifuge load. We used 4 ml gradients.
**Affinity purification**		
1 SBP tag (tested using cytosolic extracts)	None	The polysomes did not bind to streptavidin magnetic beads.
3 SBP tags (tested using cytosolic and total lysates)	Reporter polysomes bind to both magnetic and sepharose streptavidin beads	Much better binding than 1 SBP, as shown in ref 15. The polysomes cannot be eluted using biotin.
2 SBP tags	Not tested; might give sufficient binding but preserve elutability with biotin.	
Streptavidin-magnetic beads	We thought that these would give less trapped material.	More expensive than streptavidin-sepharose. In several experiments, the eluted *CAT:TUB* RNA ratio was not as high as for the sepharose beads. It might be possible to improve this by trying alternative binding and wash conditions.
Streptavidin-Sepharose beads	Usually gave about 2-fold higher *CAT:TUB* ratios than magnetic beads. Also cheaper.	Possible trapping of contaminants within the bead matrix.
Volume of sepharose beads: 25 µl for polysomes from 7 x 10^8 cells	25µl gave less contamination with *TUB* mRNA than 50µl or 75 µl.	50µl and 75 µl pulled down rather more *CAT* mRNA (about 50%) but with twice as much *TUB* contamination.
3-6 washes	3 washes were used in initial trial, but we increased it to 6 for mass spectrometry,	Additional washes are unlikely to reduce contamination much further.
Elution with 10 mM biotin	Should be specific, leaving trapped contaminants - inlcuding other polysomes - on the beads.	Nothing was eluted.
Elution with RNase	Should result in selective release of proteins that are bound to the beads via the RNA. Contamination non-polysomal components should be reduced.	In a trial using the *HSP70* 3'-UTR, no ZC3H11 was eluted. Further testing of this might be worthwhile.
Elution with SDS at 95°C	RNA and proteins released	Indiscriminate: all contaminants are obtained. The eluate is unsuitable for any further purification steps that require intact protein structure. Purification methods that require intact

		RNA secondary structure might also be affected.
Elution with alternative (partially) denaturing agents	Not tested, but could be useful if a second purification step is to be applied.	
Add a second affinity purification step	This should give sufficiently pure mRNPs to find specific bound proteins.<br>If used after our method, the additional method would have to be able to cope with mRNPs that are at least partially denatured. UV cross-linking prior to cell lysis would be essential.<br>If used before our method, the additional method would have to leave polysomes intact.	Starting with 4 x 10^9 cells, our method did not allow detection of a specific mRNP protein by mass spectrometry. This suggests that a minimum of 10^{10} cells should be used (12x 12-14 ml sucrose gradients). Assuming 5% yield from the second step, at least 10^{11} cells would be needed as starting material - nearly 100 sucrose gradients.<br>Adding a second method without intermediate storage would require working through the night.
